# Emergence of a clinical *Salmonella enterica* serovar 1,4,[5], 12: i:-isolate, ST3606, in China with susceptibility decrease to ceftazidime-avibactam carrying a novel *bla*_CTX-M-261_ variant and a *bla*_NDM-5_

**DOI:** 10.1007/s10096-024-04765-3

**Published:** 2024-02-22

**Authors:** Jie Wei, Shimei Shen, Qinghuan Zhang, Jinping Lu, Shenglan Mao, Chunhong Zou, Hua Zhou, YeLin Wei, Xingyi Ou, Jinyu Huang, Deqiang Wang, Xiaobin Li, Qun Wan, Baoju Shan, Zhenlin Zhang

**Affiliations:** 1 Department of Clinical Laboratory, Zhuhai People’s Hospital (Zhuhai Clinical Medical College of Jinan University), Zhuhai, China; 2 Key Laboratory of Molecular Biology for Infectious Diseases (Ministry of Education), Institute for Viral Hepatitis, Department of Infectious Diseases, The Second Affiliated Hospital, Chongqing Medical University, Chongqing, China; 3 Department of Clinical Laboratory, The Second Affiliated Hospital of Chongqing Medical University, Yuzhong, Chongqing, China; 4 The First People’s Hospital of Xiaoshan Hangzhou, Hangzhou, China; 5 Zhuhai Precision Medical Center, Zhuhai People’s Hospital (Zhuhai Hospital Affiliated With Jinan University), Zhuhai, China; 6 Department of Clinical Laboratory, The Fifth Affiliated Hospital of Sun Yat-Sen University, Zhuhai, China; 7 Pediatric Research Institute; Ministry of Education Key Laboratory of Child Development and Disorders; National Clinical Research Center for Child Health and Disorders (Chongqing); China International Science and Technology Cooperation Base of Child Development and Critical Disorders, Children’s Hospital of Chongqing Medical University, Chongqing, China; 8 Chongqing Key Laboratory of Pediatrics, Children’s Hospital of Chongqing Medical University, Chongqing, China

**Keywords:** Ceftazidime-avibactam, *Salmonella enterica* serovar 1,4,[5], 12: i: -, *Bla*_CTX-M-261_, *Bla*_NDM-5_, IncI1-I(α)

## Abstract

**Purpose:**

The detection rate of *Salmonella enterica* serovar 1,4,[5], 12: i: - (S. 1,4,[5], 12: i: -) has increased as the most common serotype globally. A *S*. 1,4,[5], 12: i: - strain named ST3606 (sequence type 34), isolated from a fecal specimen of a child with acute diarrhea hospitalized in a tertiary hospital in China, was firstly reported to be resistant to carbapenem and ceftazidime-avibactam. The aim of this study was to characterize the whole-genome sequence of *S.* 1,4,[5], 12: i: - isolate, ST3606, and explore its antibiotic resistance genes and their genetic environments.

**Methods:**

The genomic DNA of *S.* 1,4,[5], 12: i: - ST3606 was extracted and performed with single-molecule real-time sequencing. Resistance genes, plasmid replicon type, mobile elements, and multilocus sequence types (STs) of ST3606 were identified by ResFinder 3.2, PlasmidFinder, OriTfinder database, ISfinder database, and MLST 2.0, respectively. The conjugation experiment was utilized to evaluate the conjugation frequency of pST3606-2. Protein expression and enzyme kinetics experiments of CTX-M were performed to analyze hydrolytic activity of a novel CTX-M-261 enzyme toward several antibiotics.

**Results:**

Single-molecule real-time sequencing revealed the coexistence of a 109-kb IncI1-Iα plasmid pST3606-1 and a 70.5-kb IncFII plasmid pST3606-2. The isolate carried resistance genes, including *bla*_NDM-5_, *sul1*, *qacE*, *aadA2*, and *dfrA12* in pST3606-1, *bla*_TEM-1B_, *aac*(3)-*lld*, and *bla*_CTX-M-261_, a novel *bla*_CTX-M-1_ family member, in pST3606-2, and *aac*(6')-*Iaa* in chromosome. The *bla*_CTX-M-261_ was derived from *bla*_CTX-M-55_ by a single-nucleotide mutation 751G>A leading to amino acid substitution of Val for Met at position 251 (Val251Met), which conferred CTX-M increasing resistance to ceftazidime verified by antibiotics susceptibility testing of transconjugants carrying pST3606-2 and steady-state kinetic parameters of CTX-M-261. pST3606-1 is an IncI1-α incompatibility type that shares homology with plasmids of pC-F-164_A-OXA140, pE-T654-NDM-5, p_dm760b_NDM-5, and p_dmcr749c_NDM-5. The conjugation experiment demonstrated that pST3606-2 was successfully transferred to the *Escherichia coli* recipient C600 with four modules of OriTfinder.

**Conclusion:**

Plasmid-mediated horizontal transfer plays an important role in *bla*_NDM-5_ and *bla*_CTX-M-261_ dissemination, which increases the threat to public health due to the resistance to most β-lactam antibiotics. This is the first report of *bla*_CTX-M-261_ and *bla*_NDM-5_ in *S.* 1,4,[5], 12: i: -. The work provides insights into the enzymatic function and demonstrates the ongoing evolution of CTX-M enzymes and confirms urgency to control resistance of *S*. 1,4,[5], 12: i: -.

**Supplementary Information:**

The online version contains supplementary material available at 10.1007/s10096-024-04765-3.

## Introduction

Since *Salmonella enterica* serovar 1,4,[5], 12: i:-(*S.* 1,4,[5], 12: i:-) was reported in Spain in 1997 [1], the detection rate has increased and in recent years has surpassed *Salmonella enterica* serovar typhimurium (STM) as the most common serotype globally [2]. *S.* 1,4,[5], 12: i:—has high genetic similarity with STM, and it is speculated that 1,4,[5], 12: i:-may be a monophasic variant of phase I of STM. *S.* 1,4,[5], 12: i:-was deficient in fljB gene, which is a target site of multiplex quantitative PCR for distinguishing *S.* 1,4,[5], 12: i:-and STM [3]. Carbapenem has been considered as the last drug against most of multi-drug–resistant *Enterobacteriaceae*, including *Salmonella* spp., and remains a major public health problem [4]. So far, carbapenemase found in *Salmonella* strains includes KPC-2 [5, 6], NDM-1 [4], NDM-5 [7–9], and VIM-1 [10]. The mobile colistin resistance (mcr) genes are now also identified in different *Salmonella* serovars, including monophasic *S.* Typhimurium [11]. Plasmids carrying *bla*_NDM-5_ are generally IncFII, IncX3, IncN, and IncF [7], which lead to horizontal transmission among the same and different microorganisms. Here, we report sequence characteristics of carbapenem and ceftazidime-avibactam–resistant monophasic *S.* Typhimurium isolate, recovered from a Chinese hospital with the *bla*_CTX-M_ variant (*bla*_CTX-M-261_) and *bla*_NDM-5_ gene located on two specific transmissible plasmids, and their mobile genetic elements (MGEs). MGEs are closely associated with the formation and spread of antibiotic resistance genes (ARGs), including insertion sequences (IS), transposons, integrons, plasmids, and genomic islands [12]. Therefore, we investigated the whole genome sequence of *S.* 1,4,[5], 12: i:-ST3606, to determine the related antibiotic resistance genes and their genetic environments, especially the mobile genetic elements associated with the ARGs. In addition, we characterized the hydrolytic activity of the novel CTX-M enzyme (CTX-M-261), which differed from CTX-M-55 by an Val251Met substitution, which increased hydrolytic activities toward ceftazidime and cefepime at the expense of hydrolytic activity to cefotaxime.

## Methods

### Bacterial isolation

In September 12, 2021, at the Clinical Microbiology Laboratory of Zhuhai hospital affiliated with Jinan University (Zhuhai, Guangdong, China), one *Salmonella enterica* serovar 1,4,[5], 12: i:-strain was obtained from a stool specimen of a boy (2 years old). The outpatient was suffering from a diarrhea of 6 days (2–8 times daily) and a fever of 38.5–38.9 ℃. Before admission, antibiotic treatment (intravenous infusion of ceftazidime for 4 days and then ceftriaxone for 2 days (700 mg intravenously q12h)) was initiated, but the fever persisted. A carbapenem-resistant *Salmonella* isolate was discovered from his stool sample (designated ST3606). According to antibiotics susceptibility test result obtained by Vitek® 2 Automated Susceptibility System, the patient was then given trimethoprim-sulfamethoxazole (300 mg orally q12h). His conditions were improved after continuous antibiotic treatment and then he was discharged home after 15 days in hospital. Ethics committee approval of this study was obtained from the institutional review board of Zhuhai hospital affiliated with Jinan University, and informed consent from the patient was also obtained (code:【2022】No.51).

### Confirmation and antibiotics susceptibility testing

The species of this strain was identified using Vitek® MS (matrix-assisted laser desorption ionization-time of flight mass spectrometry, bioMérieux, Marcy-l’Étoile, France) and confirmed by single-molecule real-time (SMRT) sequencing. Serotyping of this isolate revealed 1, 4, [5], 12: i:-(Diagnostic Serum Kit, Tianrun Bio-Pharmaceutical Co. Ltd, Ningbo) confirmed by multiplex PCR [3], a monophasic variant of *S. enterica* serovar Typhimurium, which was recognized as an emerging cause of infection worldwide. Antimicrobial susceptibility was performed using the broth microdilution method, which employed the following antimicrobial agents: piperacillin, cefotaxime, cefotaxime-clavulanic acid, ceftazidime, ceftazidime-clavulanic acid, ceftazidime-avibactam, ceftriaxone, cefepime, imipenem, and meropenem, and the results were interpreted according to the Clinical and Laboratory Standards Institute (CLSI M100–S32) (CLSI, 2022). Disk diffusion method was performed for cefiderocol susceptibility. Piperacillin, ceftriaxone, imipenem, ceftazidime, clavulanic acid, and avibactam were purchased from Sigma-Aldrich, St. Louis, MO, USA. Cefepime, cefotaxime, and meropenem were purchased from MedChemExpress, New Jersey, USA. Disks of cefiderocol (30 μg) were purchased from Liofilchem, Roseto degli Abruzzi, Italy. For ceftazidime-avibactam MIC evaluation, avibactam was tested at a fixed concentration of 4 mg/L, while ceftazidime was added at different concentrations that ranged from 0.0625 to 128 mg/L. MICs were determined in triplicate on three separate days. *Escherichia coli* TOP10 (pHSG396) and *E. coli* C600 were used as quality control strains.

### Whole-genome sequencing and annotation

The genomic DNA of *Salmonella enterica* serovar 1,4,[5], 12: i:-ST3606 was extracted using NucleoBond® HMW DNA kit (MACHEREY–NAGEL, Germany) and SMRT sequencing was performed using Illumina Novaseq 6000 (Illumina, San Diego, USA) and PacBio sequencer (Suzhou Genewiz Biotechnology Co. Ltd., Suzhou, China). Raw data generated using short-read technology were optimized by the software cutadapt (v1.9.1) to generate clean data by removing adapters as well as low-quality sequences. PacBio reads were assembled using HGAP 4.0/Falcon 0.3 (Celera Assembler 8.2) [13]. Assembly polishing was performed with Pilon (version 1.22) using Illumina reads. Annotation of the ST3606 genome was completed using the National Center for Biotechnology Information (NCBI) prokaryotic annotation pipeline (PGAP). ResFinder 3.2 [14], PlasmidFinder [15], OriTfinder database [16], ISfinder database [17], and MLST 2.0 [18] were utilized to detect resistance genes, plasmid replicon type, mobile elements, and multilocus sequence types (STs) of ST3606, respectively. Sequence comparisons, map generation, and plasmid circular representation diagram were performed using BLAST (version BLAST + 2.11.0), Easyfig (version 2.2.5), and BLAST Ring Image Generator, respectively.

### Cloning of bla_CTX-M_ variants

The full lengths of the *bla*_CTX-M-1/-55/-261_ genes were synthesized and ligated to the vector pET28a and pHSG396 by BGI Genomics Co., Ltd, to generate CTX-M-1/-55/-261-pET28a and CTX-M-1/-55/-261-pHSG396 respectively. The correct constructs were confirmed by Sanger sequencing and transformed into *E. coli* TOP10 treated with 100 mM CaC1_2_ and subjected to heat-shock at 42° for 1 min. Antimicrobial susceptibilities of these constructs were determined as described above. The empty pHSG396 plasmid was used as a control.

### CTX-M-261 β-lactamase production and steady-state kinetic parameters

The recombinant CTX-M-1/-55/-261-pET28a plasmids were transformed into *E. coli* BL21 Rosetta-gami™ DE3 and grown in LB medium containing 50 mg/L kanamycin at 37 ℃ until an optical density of 0.4–0.6 (OD600) was reached. Next, 0.2 mM IPTG (isopropyl-β-d-thiogalactoside) was added and the temperature was lowered to 20 ℃ and allowed to incubate for 22 h. Cells were then harvested, resuspended in ice-cold buffer A (10 mM imidazole, 10 mM sodium phosphate, pH 7.4, and 300 mM NaCl), and then lysed by sonication in an ice-bath. The cell pellet was removed by centrifugation steps at 12,000* g* for 30 h at 4 ℃, the supernatant was filtered, and the resulting soluble fraction applied to HisTrap™ HP column (GE Healthcare) prebalanced by buffer A. After washing with buffer B (60 mM imidazole, 10 mM sodium phosphate pH 7.4, and 300 mM NaCl), the protein was eluted from the resin with buffer C (500 mM imidazole, 10 mM sodium phosphate, pH 7.4, and 300 mM NaCl). Finally, the eluted protein was loaded into a dialysis bag and was dialyzed with buffer D (10 mM sodium phosphate, pH 7.4, and 10 mM NaCl) overnight for desalination and removing imidazole. The purity of the protein was estimated to be higher than 95% by SDS-PAGE. The concentrations were determined by Pierce™ BCA Protein Assay Kit.

Kinetic parameters of cefotaxime, ceftazidime, ceftriaxone, cefepime, imipenem, and meropenem were determined using purified CTX-M-1/-55/-261 β-lactamases in 50 mM sodium phosphate buffer (pH 7.0) at 30 ℃. BSA at 20 mg/L was added to the dilute solution of CTX-M to prevent denaturation. The real-time absorbances of meropenem (298 nm), imipenem (297 nm), ceftazidime (257 nm), cefepime (254 nm), cefotaxime (257 nm), and ceftriaxone (240 nm) were determined under initial-rate conditions with a SHIMADZU UV2550 spectrophotometer (Kyoto, Japan) for 5 min. The initial velocities versus substrate concentrations were measured at least thrice. The molar extinction coefficients for tested substrates were obtained from a previous study: nitrocefin (Δ_ε482 nm_ = 15,000 M^−1^ cm^−1^), ceftriaxone (Δ_ε240 nm_ =  − 10,351 M^−1^ cm^−1^), cefotaxime (Δ_ε257 nm_ =  − 7,500 M^−1^ cm^−1^), cefepime (Δ_ε254 nm_ =  − 10,000 M^−1^ cm^−1^), ceftazidime (Δ_ε257 nm_ =  − 9000 M^−1^ cm^−1^), imipenem (Δ_ε297 nm_ =  − 9000 M^−1^ cm^−1^), and meropenem (Δ_ε298 nm_ =  − 6500 M^−1^ cm^−1^) [19, 20]. Notably, the estimated molecular weight of the tag-free CTX-M enzyme, as determined using the ExPASy-Compute pI/MW tool, approximated 28 kDa, and the isoelectric point, was determined at 7.8. The *k*_cat_ and *K*_m_ values were calculated with the GraphPad Prism 8.4 (San Diego, USA) using the Michaelis–Menten equation as follows [19, 21]:

$$\text{E}+\text{S}\begin{array}{c}k_{+1}\\\Leftrightarrow\\k_{-1}\end{array}\mathrm{ES}\xrightarrow{k_{+2}}{\mathrm E\mathrm S'}\xrightarrow{k_{+3}}\mathrm{ES}''\rightarrow\rightarrow\rightarrow\xrightarrow{k_{+n}}\text{E}+\text{P}$$

Cefotaxime and ceftriaxone behave as the best substrates of CTX-M, and the steady-state kinetic parameters, *K*m and *k*cat, were determined by Hanes–Woolf linearization or by non-linear least-square fit of the Michaelis–Menten equation [19, 21]. The reaction is described by the model: *S*_0_/*v* = (*K*_*m*_ + *S*_0_)/*V*, where *V* is the maximum rate, observed when *S*_0_ >  > *K*_m_ (substrate saturation). For ceftazidime and cefepime that is rather stable to the action of the CTX-M, with imipenem acting as transient inactivator and meropenem acting as inactivator, the *K*m value of ceftazidime and cefepime and the *K*cat value of four drugs were determined from experiments involving competition between the poor substrate and 100 μM of nitrocefin as a reporter substrate at 482 nm [21, 22].

### Western blotting

The expression levels of CTX-M-1, CTX-M-55, and CTX-M-261 in *E. coli* BL21 and *E. coli* TOP10 were determined by His-antibody. Briefly, *E. coli* carrying CTX-M-1/-55/-261-pET28a was self-induced without IPTG, and then recovered and resuspended in B-PER buffer (Thermo Scientific, MA, USA). The 30-μg total protein was subjected to SDS-PAGE, transferred onto a PVDF membrane, and probed with an His-antibody to determine the protein levels of the three CTX-M enzymes in the same *E. coli* strains.

### Conjugation experiment

The agar mating method was used to transfer β-lactam resistance to the rifampin-resistant *E. coli* C600 recipient. ST3606 and *E. coli* C600 with a McFarland standard value of 2.0 were mixed 1:1, and then the mixture dropped to the membrane placed on the solid LB medium without antibiotics, which was incubated at 37 ℃ for 18–24 h. After swirling the filtration membrane with 2 mL of liquid LB medium without antibiotics, 20 µL suspension was seeded on LB plate containing 100 mg/L ampicillin (Genview Co., Beijing, China) and 750 mg/L rifampin (Sangon Biotech Co., Shanghai, China) and cultured for 24 h. The selected transconjugant colonies were identified by PCR targeting the *bla*_CTX-M_ gene and sequencing.

### Nucleotide sequence accession numbers

The nucleotide sequences of genome, pST3606-1, pST3606-2, pST3606-3, pST3606-4, and pST3606-5 plasmids were deposited in the GenBank under the accession numbers CP094332, CP094333, CP094334, CP094335, CP094336, and CP094337, respectively. The nucleotide sequences of *bla*_CTX-M-261_ were deposited in the GenBank under the accession number OQ942222.

## Results

### Antibiotic resistance profiles of Salmonella enterica serovar 1,4,[5], 12: i:-ST3606 and conjugant

**Fig. 1:**
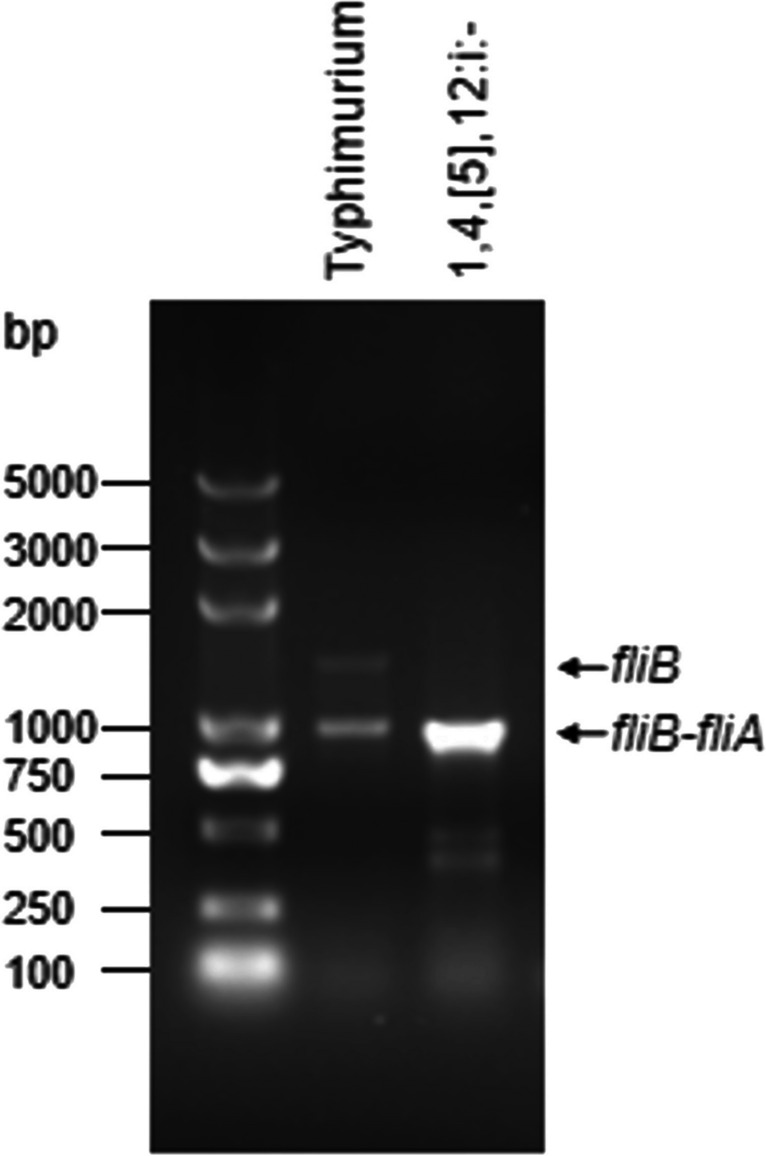
Differentiation of diphasic *Salmonella Typhimurium* and monophasic *Salmonella Typhimurium* (*S*. 1,4,[5], 12: i: -). A 1389-bp product from *Salmonella Typhimurium* that possesses a phase 2 flagellar antigen and no product from *S*. 1,4,[5], 12: i:—that lacks a phase 2 flagellar antigen

We isolated the strain and identified it as a *Salmonella enterica* serovar by MALDI-TOF MS and Diagnostic Serum Kit. We further confirmed the serotype of the strain as 1,4,[5], 12: i:-by polymerase chain reaction for amplification of fliB and fliB-fliA as described previously [3]. The result showed that only one band at 1000 bp (*fliB-fliA*) but none of band at 1389 bp (*fliB*) was found, which verified this strain was *Salmonella enterica* serovar 1,4,[5], 12: i: -, a monophasic variant of *S. enterica serovar Typhimurium* (Fig. 1)*.* Based on the results of the antibiotic susceptibility test, ST3606 exhibited resistance to most of the commonly used antibiotics, including piperacillin (> 2048 μg/mL), cefotaxime (2048 μg/mL), cefotaxime-clavulanic acid (> 128 μg/mL), ceftazidime (> 128 μg/mL), ceftazidime-clavulanic acid (> 128 μg/mL), ceftazidime-avibactam (> 128 μg/mL), ceftriaxone (> 128 μg/mL), cefepime (> 128 μg/mL), imipenem (16 μg/mL), and meropenem (32 μg/mL) (Table 1), except for levofloxacin (≤ 0.125 μg/mL), amikacin (≤ 2 μg/mL), and trimethoprim-sulfamethoxazole (≤ 20 μg/mL) (Table S1).

**Table 1:** MICs for the clinical isolate ST3606, the corresponding *E. coli* C600 transconjugant carrying *bla*_CTX-M-261_, and CTX-M-producing *E. coli* TOP10 clones

Antibiotics^a^	MIC (μg/mL)							CLSI resistance breakpoint
	*S. enterica* ST3606	*E. coli* C600 (pST3606-2)^b^	*E. coli* TOP10 (CTX-M-1-pHSG396)	*E. coli* TOP10 (CTX-M-55-pHSG396)	*E. coli* TOP10 (CTX-M-261-pHSG396)	*E. coli* TOP10 (pHSG396)	*E. coli* C600	
PRL	> 2048	> 2048	256	256	256	0.25	0.25	≥ 128
CTX	2048	64	32	128	32	≤ 0.0625	≤ 0.0625	≥ 4
CTX-CLA	> 128	64	0.25	0.125	≤ 0.0625	≤ 0.0625	≤ 0.0625	NA^c^
CAZ	> 128	64	16	32	128	≤ 0.0625	≤ 0.0625	≥ 16
CAZ-CLA	> 128	64	16	32	4	≤ 0.0625	≤ 0.0625	NA
CAZ-AVI	> 128	0.5	0.125	0.125	0.5	≤ 0.0625	≤ 0.0625	≥ 16
CRO	> 128	128	128	> 128	64	≤ 0.0625	≤ 0.0625	≥ 4
FEP	> 128	32	4	16	4	≤ 0.0625	≤ 0.0625	≥ 16
IPM	16	0.25	0.25	0.25	0.125	≤ 0.004	≤ 0.004	≥ 4
MEM	32	0.0156	0.0078	0.0078	0.0156	≤ 0.004	≤ 0.004	≥ 4

^a^*PRL*, piperacillin; *CTX*, cefotaxime; *CLA*, clavulanic acid; *CAZ*, ceftazidime; *CRO*, ceftriaxone; *FEP*, cefepime; *IPM*, imipenem; *MEM*, meropenem; *AVI*, avibactam

^b^pST3606-2 plasmid carrying *bla*_CTX-M-261_

^c^*NA*, breakpoint criterion was not available in the CLSI interpretive standards

### Overview of the Salmonella enterica serovar 1,4,[5], 12: iST3606

Whole-genome sequencing and MLST 2.0 tool analysis showed *Salmonella enterica* serovar 1,4,[5], 12: i:- ST3606 belonged to sequence type 34 (ST34). *Salmonella enterica* serovar 1,4,[5], 12: i:-ST3606 contained one chromosome and five plasmids (pST3606-1 to pST3606-5). The chromosome was 4,959,696 bp in size and contained 4769 genes with a guanine–cytosine content of 52.16%. pST3606-1 to pST3606-5 plasmids were 109,070 bp, 70,455 bp, 3592 bp, 4059 bp, and 3001 bp in size, respectively. ARGs were located on the chromosome, pST3606-1 and pST3606-2, but not on pST3606-3/-4/-5. Only one ARG coding for aminoglycoside resistance (*aac(6’)-laa*) was identified on the chromosome. Five ARGs, including *bla*_NDM-5_ coding for β-lactam resistance, *sul1* coding for sulfonamide resistance, *qacE* coding for antiseptic resistance, *aadA2* coding for aminoglycoside resistance, and *dfrA12* coding for trimethoprim resistance, and three ARGs, including *bla*_CTX-M-261_ and *bla*_TEM-1B_ coding for β-lactam resistance and *aac(3)-lld* coding for fluoroquinolone/aminoglycoside resistance, were identified on pST3606-1 and pST3606-2 plasmids, respectively (Table 2). *bla*_NDM-5_ has been reported in *Salmonella enterica* serovar 1,4,[5], 12: i:-for the first time in this article, although it was previously reported in *Salmonella enterica* serovar Typhimurium. Interestingly, we also found a novel *bla*_CTX-M_ gene on another plasmid. According to their genetic locations, the ARGs were divided into three drug-resistance regions, for which the genetic structures are described in the following sections. Through BLAST analysis and the GenBank database, we detected identical or similar sequences in other bacteria.

**Table 2:** Genomic characteristics of *Salmonella enterica* serovar 1,4,[5], 12: i:—isolate ST3606

Genetic material	Plasmid type	Size (bp)	GC content (%)	Antimicrobial resistance gene (s)
Chromosome	/	4,959,696	51%	*aac(6’)-laa*
pST3606-1	IncI1-1(α)	109,070	50%	*bla*_NDM-5_, *sul1*, *qacE*, *aadA2*, and *dfrA12*
pST3606-2	IncFII	70,455	52%	*bla*_CTX-M-261_, *bla*_TEM-1B_, and *aac(3)-lld*
pST3606-3	/	3592	43%	/
pST3606-4	/	4059	49%	/
pST3606-5	/	3001	44%	/

### Genetic contexts associated with bla_NDM-5_ and class I integron

**Fig. 2:**
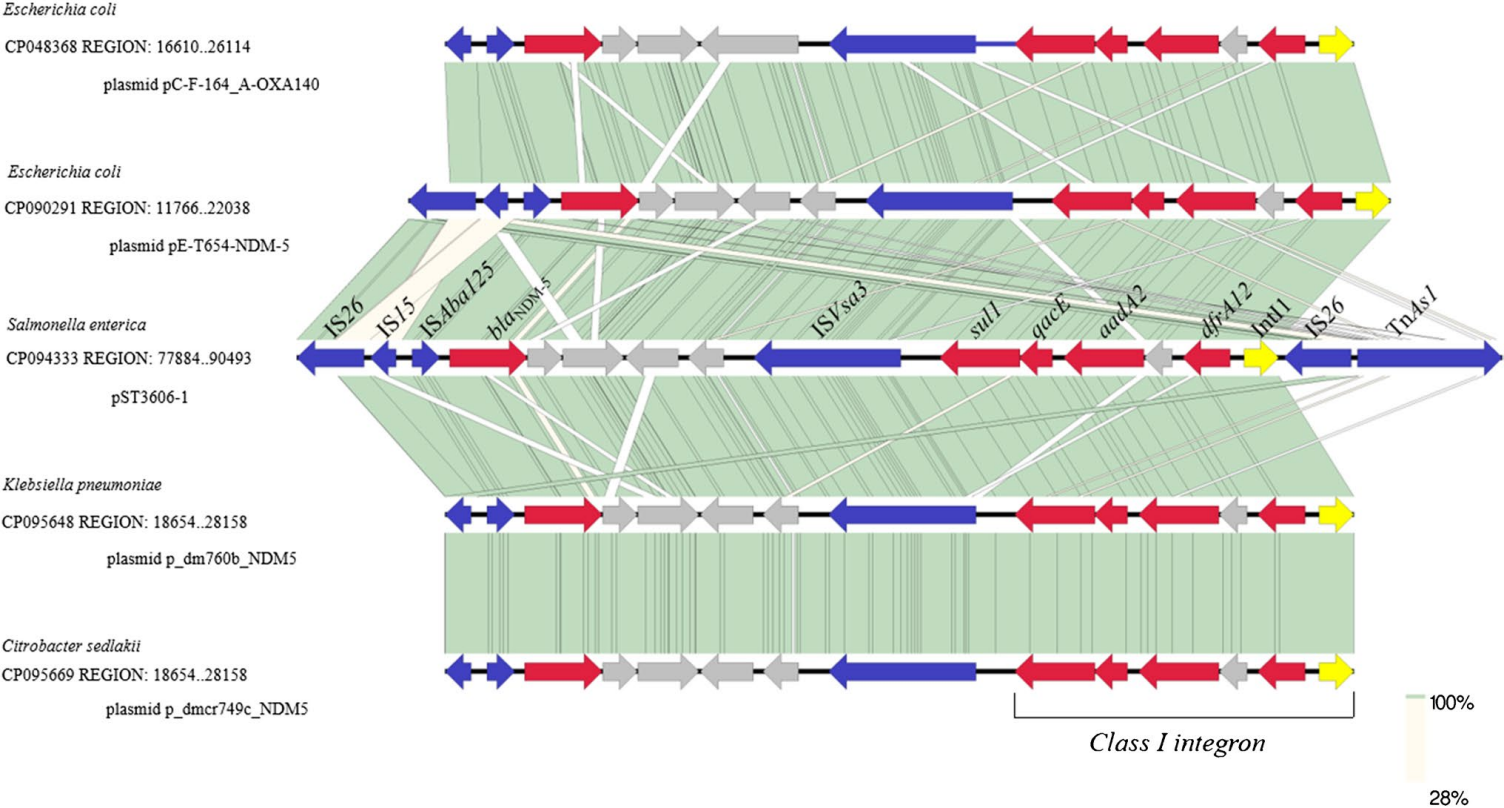
A 10.9-kb IncI1-α sequence of the genetic context of the NDM-5-harboring plasmid pST3606-1 is shown. Linear genetic structure comparison of plasmid pST3606-1 (*Salmonella Typhimurium* (*S*. 1,4,[5], 12: i: -), GenBank accession number CP094333), pC-F-164_A-OXA140 (*Escherichia coli*, GenBank accession number CP048368), pE-T654-NDM-5 (*Escherichia coli*, GenBank accession number CP090291), p_dm760b_NDM-5 (*Klebsiella pneumoniae*, GenBank accession number CP095648), and p_dmcr749c_NDM-5 (*Citrobacter sedlakii*, GenBank accession number CP095669). The arrows indicate open reading frames. The red arrows represent antibiotic resistance gene. The blue arrows represent the transposon and IS elements. The yellow arrows represent Integrase gene. Light green shading denotes homology regions. The depth of shadowing is indicative of the BLASTn matching degree. IS, insertion sequences. Color images are available online. Comparisons performed using a nucleotide basic local alignment sequence tool search and visualized using in silico molecular cloning genomics edition software

*Salmonella enterica* serovar 1,4,[5], 12: i:-ST3606 contained *bla*_NDM-5_, which encoded for New Delhi metallo-β lactamase that hydrolyzes almost all β-lactam antibiotics including carbapenem. The *bla*_NDM-5_ gene was located on the coordinate 79,483-80,295 of pST3606-1 with a GC content of 50.6%, which was identified as a typical IncI1-I(α) plasmid containing regions involved in plasmid stability, replication, and conjugative transfer (Fig. 2). The *bla*_NDM-5_ was embedded in an IS*26*, IS*15*, and *Aba125*-mediated transposition unit, with IS*26*, IS*15*, and IS*Aba125* located upstream of *bla*_NDM-5_, forming the genetic structure “IS*26*-IS*15*-IS*Aba125-bla*_NDM-5_” (77,884-80,295), which has also been found in diverse isolates in different genetic contexts, including the *E. coli* plasmid pC-F-164_A-OXA140 (91% query coverage and 99.98% identity, GenBank accession no. CP048368), *E. coli* plasmid pE-T654-NDM-5 (100% query coverage and 99.98% identity, GenBank accession no. CP090291), the *Klebsiella pneumoniae* plasmid p_dm760b_NDM-5 (96% query coverage and 99.98% identity, GenBank accession no. CP095648), and *Citrobacter sedlakii* plasmid p_dmcr749c_NDM-5 (100% query coverage and 99.96% identity, GenBank accession no. CP095669) (Fig. 2). The *sul1*, *qacE*, *aadA2*, and *dfrA12* genes, which mediated sulfonamide, disinfectant, aminoglycoside, and trimethoprim resistance, were located upstream of integrase type I (*Intl1*) and on the coordinate 84,608-85,447; 85,507-85,788; 85,952-86,743; and 87,151-87,648, respectively. The class I integron of *Salmonella enterica* serovar 1,4,[5], 12: i:-ST3606 was 3547 bp in size (coordinate: 84,608–88,155) and contained the trimethoprim resistance gene *dfrA12*, aminoglycoside resistance gene *aadA2*, disinfectant resistance gene *qacE*, trimethoprim resistance gene *sul1*, and integrase (intI1). We found that IS*Vsa3* was located upstream of class I integron followed by the IS26 and Tn*As1* transposons, forming a typical Tn*As1* transposition unit (Fig. 2).

### Genetic contexts associated with bla_CTX-M-261_

**Fig. 3:**
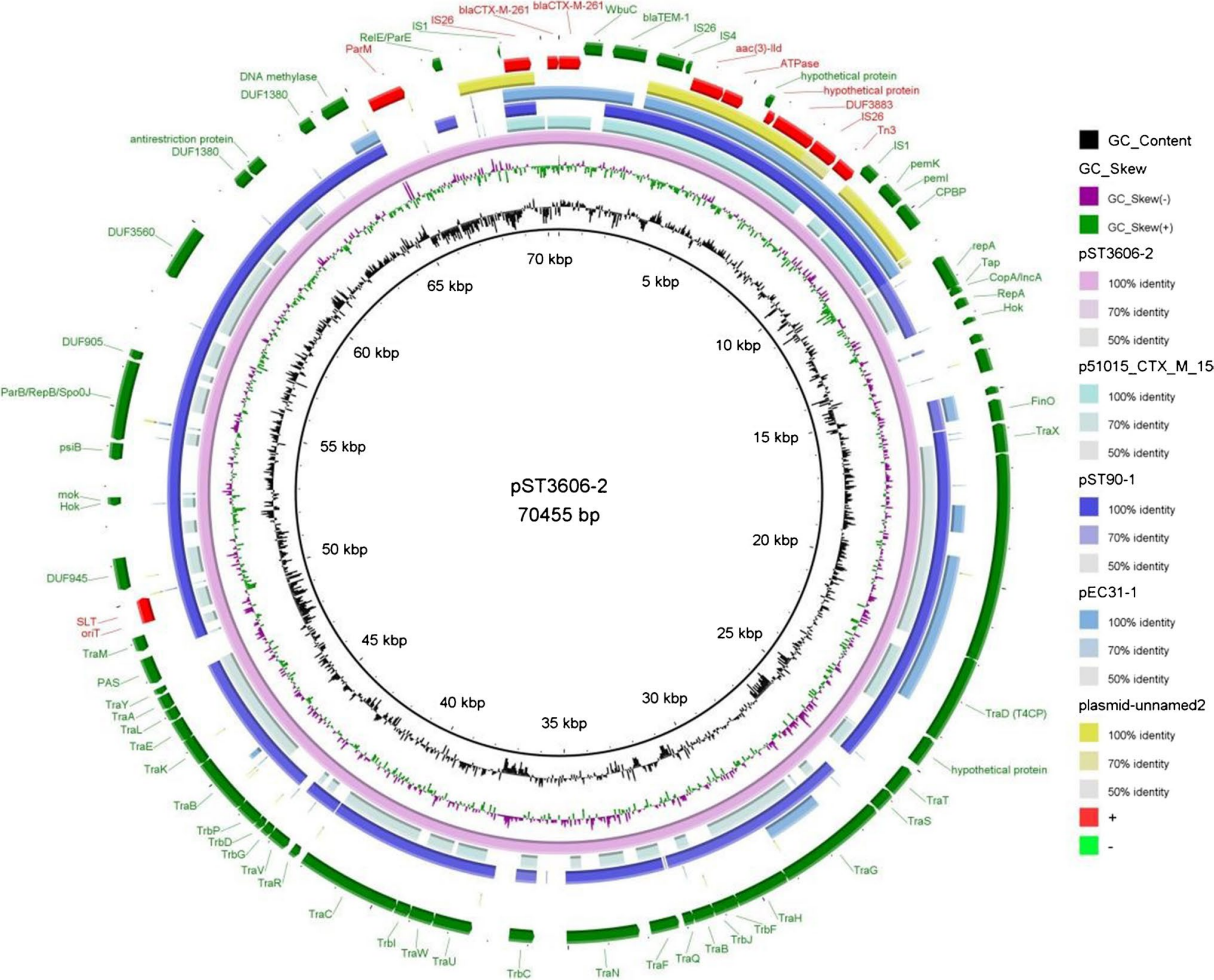
Circular representation of pST3606-2. The innermost circle represents GC content; the second inner circle represents GC skew (green, GC + ; purple, GC-). These mcr-8-carrying plasmids pST3606-2 (*Salmonella Typhimurium* (*S*. 1,4,[5], 12: i: -), GenBank accession number CP094334), p51015_CTX_M_15 (*Klebsiella pneumoniae*, GenBank accession number CP050379), pST90-1 (*Salmonella enterica*, GenBank accession number CP050735), pEC31-1 (*Escherichia coli*, GenBank accession number CP056037), and plasmid unnamed 2 (*Shigella flexneri*, GenBank accession number CP058841) were represented by pink, light blue, purple, dark blue, and yellow circles, respectively. The outermost red circle depicts gene distribution of the resistance gene, transposon, IS elements, and integrative and conjugative element. *bla*_CTX-M-261_, CTX-M-261 extended spectrum β-lactamase gene; aac(3)-lld gene, aminoglycoside resistance gene; ATPase, adenosine triphosphate synthase; hypothetical protein

The IncFII-type plasmid pST3606-2 carried by ST3606 shares a similar backbone with the plasmid pST90-1 (84% query coverage and 100% identity, GenBank accession no. CP050735) which was identified in a strain of *S*. enterica but carrying *bla*_*CTX-M-27*_ isolated from a patient in the USA (Fig. 3). The main difference between pST3606-2 and pST90-1 was that pST3606-2 contained a 4941-bp complex transposon structure carrying *bla*_CTX-M-261_, a novel *bla*_CTX-M_ gene carrying a single-nucleotide mutation 751G > A leading to amino acid substitution of Val for Met at position 251 (Val251Met) on the coordinate 70,155-70,455, 1-575. *bla*_CTX-M-261_, bracketed by *IS*1 elements and *IS*4 elements, could encode extended-spectrum beta-lactamase (ESBL) conferring resistance to the extended-spectrum cephalosporins. The *bla*_CTX-M_ gene of pST3606-2 was organized as “IS*1*-IS*26*-*bla*_CTX-M-261_-WbuC-*bla*_TEM-1_-IS*26*-IS*4*” (Fig. 3), which among plasmids in the NCBI nucleotide database, IncFII plasmid was positive for *bla*_CTX-M_. The conjugation experiment demonstrated that pST3606-2 was successfully transferred from the donor strain (ST3606) to the recipient (*Escherichia coli* C600) and conferred beta-lactam but not carbapenem resistance to the recipient strain due to the pST3606-2 containing *bla*_CTX-M-261_ and *bla*_TEM-1_ genes (Table 1 and 2). The conjugation frequency of pST3606-2 was 10^–3^ per recipient cell for ST3606. We further analyzed the conjugative transfer region of the conjugative plasmid pST3606-2 using software oriTfinder, and the results showed that it contained the intact conjugative transfer region, including origin of transfer site (oriT) on the coordinate 49,414-49,499, relaxase gene on the coordinate 16,618-21,888, gene encoding type IV coupling protein (T4CP) on the coordinate 21,888-24,113, and gene cluster for Tra_F-like IV secretion system (T4SS) on the coordinate 15,852-50,067 (Fig. 3).

### Steady-state kinetics of CTX-M-261

**Fig. 4:**
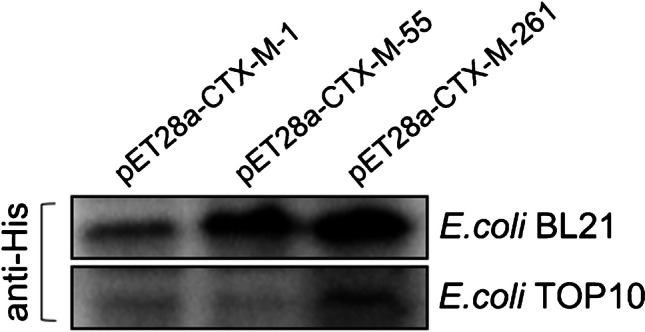
The protein expression levels of CTX-M-1/55/261 in *E. coli* BL21 and *E. coli* TOP10 were compared

The purities of CTX-M proteins were more than 95% as estimated by SDS-PAGE. CTX-M-55, differing from CTX-M-261 variant by only one single amino acid substitution, has the highest homology and was employed as a contrast enzyme. The steady-state kinetic parameters *k*_cat_ and *K*_m_ of CTX-M-1, CTX-M-55, and CTX-M-261 among a set of substrates (Table 3) indicated that all cephalosporins tested but not carbapenem could be hydrolysed by CTX-M. Cefotaxime was the best substrate for CTX-M-261, with a catalytic efficiency of 7.9 μM^−1^ s^−1^. But, for CTX-M-1 and CTX-M-55, the highest catalytic efficiency happened to ceftriaxone (9.98 μM^−1^ s^−1^ and 16.67 μM^−1^ s^−1^, respectively), which was consistent with a previous report [20]. Compared to CTX-M-55, CTX-M-261 exhibited significantly decreased affinity and diminished turnover for ceftriaxone (0.7 μM^−1^ s^−1^ vs. 16.67 μM^−1^ s^−1^), which was also confirmed by their MICs of this drug (64 μg/mL vs. > 128 μg/mL) (Table 1). Intriguingly, the *K*_m_ value of CTX-M-261 catalyzing ceftazidime could be determined, but its *k*_cat_/*K*_m_ value was also too low (0.06 μM^−1^ s^−1^). However, regarding the MICs of ceftazidime for *E. coli* TOP10 carrying *bla*_CTX-M_, CTX-M-261 mediated the highest MIC (Table 1), which seem to be in agreement with the *K*_m_ value. Sometimes, in β-lactamase-overproducing strains, very poor activities against some substrates can nonetheless lead to amazingly increased MIC values for these drugs. So, we examined the protein expression of three CTX-Ms. CTX-M-261 was significantly increased compared with CTX-M-1/-55 in the same bacterial background environment, which was not consistent with the result of He D et al. (Fig. 4). The hydrolytic activities of the three CTX-M were undetectable against imipenem and meropenem as inactivators.

**Table 3:** Kinetic parameters of CTX-M-261, CTX-M-1, and CTX-M-55

Substrate^a^	CTX-M-261			CTX-M-1			CTX-M-55		
	*K*_*m*_ (μM)	*k*_*cat*_ (s^−1^)	*k*_*cat/*_*K*_*m*_ (μM^−1^ s^−1^)	*K*_*m*_ (μM)	*k*_*cat*_ (s^−1^)	*k*_*cat/*_*K*_*m*_ (μM^−1^ s^−1^)	*K*_*m*_ (μM)	*k*_*cat*_ (s^−1^)	*k*_*cat/*_*K*_*m*_ (μM^−1^ s^−1^)
CTX	18.3 ± 1.9	144 ± 16	7.9	17.8 ± 2.5	111 ± 25	6.2	23.4 ± 4.9	123 ± 32	5.3
CAZ	166.7 ± 37.1	10.6 ± 1.1	0.06	328.8 ± 42	43.6 ± 3.7	0.13	736 ± 79.7	31.3 ± 3.1	0.04
CRO	36 ± 3.8	25.4 ± 1.3	0.7	10.5 ± 1.8	104.9 ± 4.3	9.98	6.6 ± 0.5	111 ± 3.9	16.67
FEP	124.6 ± 26.9	17.1 ± 1.5	0.13	146 ± 21.3	27.9 ± 8.6	0.19	1610.7 ± 111	5.8 ± 0.7	0.004
IPM	ND^b^	< 0.01	ND	ND	< 0.01	ND	ND	< 0.01	ND
MEM	ND	< 0.01	ND	ND	< 0.01	ND	ND	< 0.01	ND

^a^Data are the averages of the results obtained from three independent experiments. *CTX*, cefotaxime; *CAZ*, ceftazidime; *CRO*, ceftriaxone; *FEP*, cefepime; *IPM*, imipenem; *MEM*, meropenem

^b^*ND*, not determined due to a low initial rate of hydrolysis

## Discussion

*Salmonella enterica* serovar Typhimurium, especially monophasic *S. enterica* serovar Typhimurium (*S*. 1,4,[5], 12: i: -) is one of the most prevalent Nontyphoidal Salmonella (NTS) bacterial causes of gastrointestinal infections worldwide in the last two decades [23]. There have been lots of reports describing the multidrug resistance of *Salmonella* Typhimurium, including β-lactams, aminoglycosides, and colistin [24]. But there have been no reports about *S*. 1,4,[5], 12: i:-resistant to both carbapenem and ceftazidime-avibactam. We report for the first time a *S*. 1,4,[5], 12:i-strain carrying *bla*_NDM_ and *bla*_CTX-M_ genes and resistant to carbapenem and ceftazidime-avibactam. Although the *bla*_NDM_ and *bla*_CTX-M_ genes were identified in *S.* Typhimurium from pork [7, 9] and a clinical patient [8] in China, this is the first case of the occurrence of *bla*_NDM-5_-positive and *bla*_CTX-M-261_-positive, coding a novel CTX-M enzyme variant that differed from CTX-M-55 by a single amino acid substitution (Val251Met) due to one missense point mutation at position 751 (G → A), in *S*. 1,4,[5], 12: i: -. Notably, the genetic context of *bla*_NDM_ in this isolate ST3606 (IS*26*-IS*15*-IS*Aba125-bla*_NDM-5_-IS*Vsa3-sul1*-*qacE*-*aadA2*-*dfrA12-*IntI1) was different from that of pNDM5-SSH006 in *S*. Typhimurium SSH006 (IS*SWi1*-IS*3000*-ΔIS*Aba125-*IS*5-bla*_NDM-5_-*ble-trpF*-*tat*-IS*26-*Δ*umuD*). Moreover, on the downstream of *bla*_NDM-5_ on pST3606-2, we found a *IntI1* Integrase gene considered as a mobilizable platform that promotes ARG transfer and transformation in the environment and reflect the impact of human activities [25]. Surprisingly, the *E. coli* C600 transconjugant (pST3606-2) was not sensitive to cefiderocol with inhibition zones of 14 mm at 30 μg/disk according to the antimicrobial disk susceptibility tests (Zone Diameter Breakpoints: S, ≥ 16 mm; I, 9–15 mm; R, ≤ 8 mm; Figure S1). Cefiderocol, a novel siderophore-substituted cephalosporin with antibacterial activity against a broad spectrum of multidrug-resistant Gram-negative bacteria even including those that produce either KPC enzymes or NDM family, was approved by the FDA on November 2019 [26]. Hence, the analyzed genome content draws attention to the problem of multidrug-resistant *S*. 1,4,[5], 12: i:-isolated in clinic and a potential threat to human health.

The assembled genome sequences showed that ST3606 possesses a 4.96-Mb chromosome and two plasmids carrying ARGs: a 109 070-bp IncI1-Iα plasmid NDM-5 (designated pST3606-1) encoding acquired resistance genes, such as *bla*_NDM-5_, *sul1*, *qacE*, *aadA2*, and *dfrA12*, and a 70,455-bp IncFII plasmid (designated pST3606-2) encoding genes that confer resistance to β-lactams (*bla*_CTX-M-261_ and *bla*_TEM-1B_), aminoglycosides (*aac(3)-lld*) (Figs. 1 and 2). The major plasmid types carrying *bla*_NDM-5_ from reference NCBI database included IncX3 (29.68%), IncFII (15.41%), IncFIB (12.79%), and IncC (9.59%) [27]. The *bla*_NDM_ gene previously reported to be carried by *Salmonella* appears on the IncX3 (*bla*_NDM-5_), IncA/C (*bla*_NDM-1_), IncFII (*bla*_NDM-5_), IncFIB (*bla*_NDM-5_), and IncI1 (*bla*_NDM-13_) plasmids, and chromosome (*bla*_NDM-9_ and *bla*_NDM-1_) [28]; particularly, the IncX3 and IncA/C plasmids are the most prevalent. However, IncI1-Iα plasmid carrying *bla*_NDM-5_ has not appeared in *Salmonella*, only a *Salmonella* Rissen ST469 harboring IncI1 plasmid carrying *bla*_NDM-13_ [29]*.*

The wide spread of CTX-M variants among *Salmonella* isolates represents a large threat to the public health globally [30]. To date, more than 260 CTX-M variants have been named and deposited in the GenBank database. In this study, one novel *bla*_CTX-M-261_ variant, that belong to *bla*_CTX-M-1like_ group according to Ambler classification method [31], was carried by *S*. 1,4,[5], 12: i:-isolated from the patient. Compared to CTX-M-55, amino acid substitution (Val251Met) conferred CTX-M-261 enzyme higher affinity (166.7 μM vs 736 μM) with ceftazidime but not higher hydrolytic activity (0.06 μM^−1^ s^−1^ vs 0.04 μM^−1^ s^−1^) in enzyme kinetics experiment. However, MICs in the *E. coli* TOP10 clones producing CTX-M-261 were higher due to the higher expression. In addition, CTX-M-261 may be an evolution leading to development of cefiderocol susceptibility decrease [32]. We speculate the presence of cross-resistance of CTX-M-261 between cefatzidime and cefiderocol. As such, this study increases our understanding that *bla*_CTX-M_ variants are undergoing continuous evolution and thus need to be closely monitored.

WGS revealed the new variant *bla*_CTX-M-261_ was located on a conjugational IncFII-type plasmid. IncFII plasmids have been found to be associated with various resistance genes including ESBLs, and carbapenemase encoding genes in *Salmonella* [33, 34]. Complete conjugative transfer region was identified in the plasmid, which is consistent with the finding that the *bla*_CTX-M-261_ harboring IncFII-type plasmid can be transferred by conjugation [35]. It is noteworthy to mention, as shown in Table 1, that most of the antibiotic susceptibility profiles of ST3606 were consistent with *E. coli* C600 transconjugant, except for imipenem, meropenem, and ceftazidime-avibactam, which indicated that not *bla*_CTX-M-261_ but *bla*_NDM-5_ plays a dominant role in yielding to resistance of carbapenem and ceftazidime-avibactam. However, we found MIC for ceftazidime-avibactam of *E. coli* TOP10 transformant carrying *bla*_CTX-M-261_ (0.5 mg/L) is consistent with *E. coli* C600 transconjugant (0.5 mg/L) and higher than those of *E. coli* TOP10 transformant carrying *bla*_CTX-M-1/-55_ (0.125 mg/L), which speculated *bla*_CTX-M-261_ may be an evolution leading to development of ceftazidime susceptibility decrease [36].

To the best of our knowledge, this is the first report describing *bla*_NDM-5_ and a novel *bla*_CTX-M_ variant in *S*. 1,4,[5], 12: i:-isolate with susceptibility decrease of ceftazidime. On the one hand, this work extended our understanding of enzymatic function and demonstrated the ongoing evolution of CTX-M enzymes. While focusing on the evolution of NDM carbapenemase, a close surveillance of CTX-M-producing pathogens should be enacted for continued monitoring of the spread of CTX-M variants [37]. On the other hand, the comparison of pST3606-1 showed that *E*. coli, *Klebsiella pneumoniae*, and *Citrobacter sedlakii* share a complete conserved plasmid backbone (IS*26*-IS*15*-IS*Aba125-bla*_NDM-5_-IS*Vsa3-sul1*-*qacE*-*aadA2*-*dfrA12-*IntI1), which shows the prevalence of the plasmid with a strong transmissibility among different species widely [38]. Especially, it has been confirmed that persistent *Salmonella* isolates could promote the spread of antibiotic resistance plasmids in the gut [39]. Hence, identifying the mechanism of the spread of carbapenem-resistant *Salmonella* in the environment has become a substantial global health concern.

In this study, we investigated the genetic characteristics of *Salmonella enterica* Serovar 1,4,[5], 12: i:-isolate ST3606 carrying *bla*_CTX-M-261_ and *bla*_NDM-5_, and characterized steady-state kinetics of CTX-M-261. Notably, this is the first report finding the *S.*1,4,[5], 12: i:- carrying both NDM and a novel CTX-M (CTX-M-261). *S.*1,4,[5], 12: i:-is the the predominant serovar in both humans and animals in China. CTX-M-261 may be an evolution leading to development of ceftazidime susceptibility decrease. The IS element upstream and IntI1 element downstream of *bla*_NDM-5_, and the IS*26* element upstream and downstream of *bla*_CTX-M-261_ will contribute to horizontal gene transfer between different bacteria in environment. Further surveillance and increased measures should be adapted to prevent the transmission of *bla*_NDM-5_-carrying *S.*1,4,[5], 12: i:-strains and evolution of *bla*_CTX-M_ in clinic.

### Supplementary Information

Below is the link to the electronic supplementary material.

Supplementary file1 (DOCX 474 KB)

## Data Availability

Complete sequences of the chromosome and plasmids from *Salmonella enterica* serovar 1,4,[5], 12: i:—ST3606 have been deposited in GenBank under accession numbers CP094332–CP094337, respectively. The nucleotide sequences of *bla*_CTX-M-261_ were deposited in GenBank under the accession number OQ942222.
