# Supplementary material for: Emergence of a clinical Salmonella enterica serovar 1,4,[5], 12: i:-isolate, ST3606, in China with susceptibility decrease to ceftazidime-avibactam carrying a novel blaCTX-M-261 variant and a blaNDM-5
Source: Eur J Clin Microbiol Infect Dis. 2024 Feb 22;43(5):829–40. doi: 10.1007/s10096-024-04765-3 (PMC11108873; doi:10.1007/s10096-024-04765-3)
Supplement: Supplementary file 1 — Supplementary file1 (DOCX 474 KB) [file 10096_2024_4765_MOESM1_ESM.docx]

**SUPPLEMENTARY MATERIAL**

TABLE S1 MICs for the clinical isolate ST3606.

| Antibiotics^a^ |  | MIC(*μ*g/mL) | Resistance | CLSI resistance breakpoint |
| --- | --- | --- | --- | --- |
|  |  | *S. Enterica* ST3606 |  |  |
| PRL-TAZ |  | >128 | R | ≥32/4 |
| AMX-CLA |  | >32 | R | ≥32/16 |
| AMK |  | ≤2 | S | ≥64 |
| LEF |  | ≤0.125 | S | ≥2 |
| CFP-SUL |  | >64 | R | ≥64/32 |
| SXT |  | ≤20 | S | ≥4/76 |

^a^ PRL, Piperacillin; TAZ, Tazobactam; AMX, Amoxicillin; CLA, Clavulanic acid; AMK, Amikacin; LEF, Levofloxacin; CFP,Cefoperazone; SUL, Sulbactam; SXT,Trimethoprim/Sulfamethoxazole.


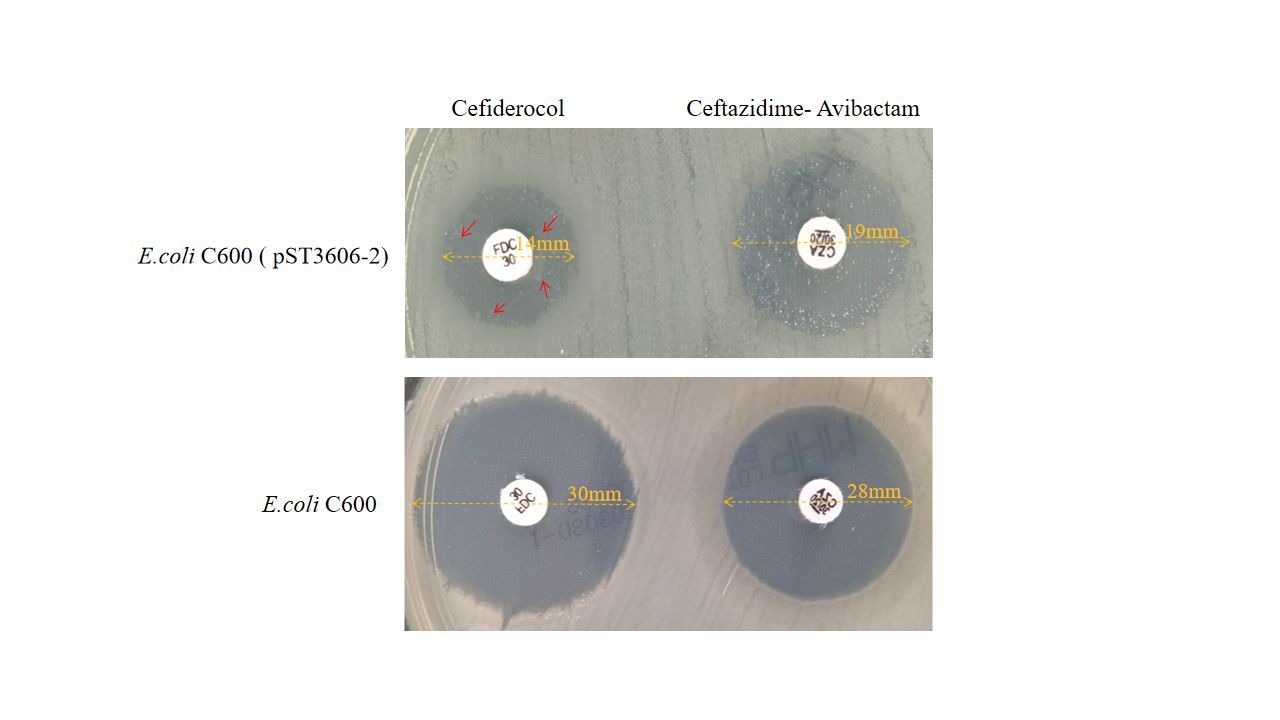


Figure S1. Zone Diameter Disk Diffusion for *E.coli* C600 transconjugant ( pST3606-2) Direct From MH Culture. Dotted yellow arrows and yellow number indicates the corresponding measurement diameter. Results were interpreted according to the Clinical and Laboratory Standards Institute (CLSI M100–S32) (CLSI, 2022). The red arrow shows colonies growing within the inhibition zone.
